# A note on heat kernel of graphs

**DOI:** 10.1016/j.heliyon.2024.e32235

**Published:** 2024-06-04

**Authors:** Yang Yang, Wei Ke, Zhe Wang, Haiyan Qiao

**Affiliations:** a College of Aerospace and Civil Engineering, Harbin Engineering University, Harbin 150001, China; b College of Artificial Intelligence, Tianjin University of Science and Technology, Tianjin 300457, China; c Key Laboratory of Dual Dielectric Power Technology, Hebei Hanguang Industry Co. Ltd., Handan 056017, China; d School of Information and Electrical Engineering, Hebei University of Engineering, Handan 056038, China

**Keywords:** 05C50, 05C09, 05C92, Heat kernel, Almost equitable partition, Principal submatrix, Laplacian matrix

## Abstract

Consider a simple undirected connected graph *G*, with D(G) and A(G) representing its degree and adjacency matrices, respectively. Furthermore, L(G)=D(G)−A(G) is the Laplacian matrix of *G*, and $H_{t}=\exp\left(-t\text{L}(G)\right)$ is the heat kernel (HK) of *G*, with t>0 denoting the time variable. For a vertex u∈V(G), the *u*th element of the diagonal of the HK is defined as $H_{t}(u,u)={\left(\exp(-t\text{L}(G))\right)}_{uu}=\sum_{k=0}^{\infty }\frac{{\left({\left(-t\text{L}(G)\right)}^{k}\right)}_{uu}}{k!}$, and $HE(G)=\sum_{i=1}^{n}e^{-t\lambda_{i}}=\sum_{u=1}^{n}H_{t}(u,u)$ is the HK trace of *G*, where $\lambda_{1},\lambda_{2},\cdots ,\lambda_{n}$ denote the eigenvalues of L(G). This study provides new computational formulas for the HK diagonal entries of graphs using an almost equitable partition and the Schur complement technique. We also provide bounds for the HK trace of the graphs.

## 1 Introduction

Assume that G=(V(G), E(G)) is a simple undirected connected graph, with E(G) and V(G) being its edge and vertex sets, respectively. Furthermore, D(G) and A(G) are the degree and adjacency matrices of *G*, $d_{u}$ is the degree of vertex *u*, and L(G)=D(G)−A(G) is the Laplacian matrix of *G*. Assuming that $\lambda_{1}\geq \lambda_{2}\geq \cdots \geq \lambda_{n}=0$ are the eigenvalues of L(G), the heat kernel (HK) of *G* [1], [2] is defined as

$$H_{t}=\exp\left(-t\text{L}(G)\right),$$

where t>0 is the time variable.

The diagonal elements of HK represent heat or diffusion at each node in the graph. Let $H_{t}(u,u)=\exp{\left(-t\text{L}(G)\right)}_{uu}$ denote the *u*th diagonal element of the HK, representing the heat or diffusion at the *u*th graph node. This provides information on the probability density of a diffusion process that starts and remains at the same node over time [3]. By examining the diagonal elements of HK, we can gain insights into the centrality or importance of the graph nodes [4], [5]. Specifically, nodes with higher diagonal elements may be considered more central or influential with respect to the heat or diffusion dynamics within the graph [6], [7]. Further studies on HK have also been conducted in physics, chemistry, and mathematics [8], [9], [10], [11].

Let $HE(G)=\sum_{i=1}^{n}\exp(-t\lambda_{i})=\sum_{u=1}^{n}H_{t}(u,u)$ be the HK trace of graph *G*. HE(G) can be used to interpret the diffusion processes and dynamics of *G*, as it provides information on the spread of heat or random walks on the graph over time [12], [13]. Research on the HK trace have also been conducted in the domains of network analysis, machine learning, and social network analysis [14], [15], [16], [17], [18], [19].

If $V_{j}$ is the neighbor cardinality of a vertex in $V_{i}$, denoted by $m_{ij}=\left|N_{G}(v)\cap V_{j}\right|$, and is independent of the selected vertex in $V_{i}$, then a *k*-partition of V(G), denoted by $\pi =\left(V_{1},\ldots ,V_{k}\right)$, is said to be equitable. Thus, the value of $m_{ij}$ only depends on *i* and *j* for any pair of subsets i,j∈{1,…,k} and vertex $v\in V_{i}$. The matrix ${\text{P}}^{\pi }$ is the characteristic matrix of *π*, with columns representing the characteristic vectors of the subsets $V_{1},\ldots ,V_{k}$.

The concept of equitable partition was introduced by [20], [21]. An equitable partition of *G* with *n* vertices is the partition $V(G)=V_{1}\cup V_{2}\cup \cdots \cup V_{k}$, where $V_{i}$ has the same neighbor cardinality in each $V_{j}$ for all i,j∈{1,2,...,k}. The constant $b_{ij}$ must satisfy this condition to form an equitable partition, with the matrix ${\left(b_{ij}\right)}_{k\times k}$ representing the divisor matrix of V(G). As the orbits of any automorphism group in *G* constitute an equitable partition, *G* has equitable partitions. In addition, the spectrum of ${\left(b_{ij}\right)}_{k\times k}$ is contained in that of *G* [22].

The quotient graph G/π of *G* associated with the equitable *k*-partition *π* is a multi-digraph whose vertices represent subsets of $\pi ,V_{1},\ldots ,V_{k}$, with $d_{ij}$ arcs ranging from $V_{i}$ to $V_{j}$. The present study is based upon an equitable partition, with an almost equitable partition defined as a *k*-partition of V(G), such that ∀i,j∈{1,…,k} with i≠j, $\forall x\in V_{i}$, $\left|N_{G}(x)\cap V_{j}\right|=d_{ij}$, and as a *k*-partition of the vertices of *G* [23], [24].

The generalized Laplacian matrix ${\left({\text{L}}^{\pi }\right)}_{k\times k}$ is defined as follows when considering an almost equitable *k*-partition *π*. Note that ${\text{L}}^{\pi }$ is the Laplacian matrix of quotient graph G/π when *π* is an equitable partition.

$${\left({\text{L}}^{\pi }\right)}_{ij}=\left\{ \begin{array}{cc} -d_{ij} & \text{ if }i\neq j,\\ s_{i} & \text{ otherwise } \end{array}\right.$$

where $s_{i}=\sum_{j\neq i}d_{ij}$.

Based on the definition provided, every equitable partition is almost equitable, although the converse does not hold true. This implies that each graph *G* contains almost equitable partitions. Equitable and almost equitable partitions have essential applications in control theory, chemical studies, and complex networks [23], [24], [25], [26], [27].

This study utilized the principal submatrix of the Laplacian matrix and almost equitable partitions to provide new equations for the HK diagonal entries of graphs. These equations can help compute the heat kernel diagonal entries of a large graph using the structures of smaller subgraphs. Furthermore, we obtained bounds for the HK trace of graphs.

The remainder of this paper is organized as follows. Section 2 details the diagonal entries of HKs in the context of the principal submatrix of the Laplacian matrix and almost equitable partitions of graphs. Section 3 provides expressions for the HK trace estimations of graphs. Finally, Section 4 concludes the study.

## 2 Calculation of heat kernel diagonal entries

Consider J, e, and I to be the all-ones matrix, all-ones vector, and identity matrix, respectively. Let $c_{i}(\text{M})$ denote the *i*-th column sum of matrix M and Tr(M) denote the trace of M.

We now present a block matrix representing $H_{t}$.

Theorem 2.1 *Let G be a connected graph with n vertices, where*$\text{L}(G)=\left(\begin{array}{cc} d_{u} & -{\text{b}}^{⊤}\\ -\text{b} & {\text{L}}_{u} \end{array}\right)$*is the Laplacian matrix. Then*

$$H_{t}=\left(\begin{array}{cc} 1+n^{-1}{\text{e}}^{⊤}(\text{M}-\text{I})\text{e} & n^{-1}{\text{e}}^{⊤}(\text{I}-\text{M})\\ \text{e}-n^{-1}\text{J}(\text{I}-\text{M})\text{e}-\text{M}\text{e} & n^{-1}\text{J}(\text{I}-\text{M})+\text{M} \end{array}\right),$$

*where*$\text{M}=\exp(-t((\text{J}+\text{I}){\text{L}}_{u}))$*,*${\text{L}}_{u}$*is the principal submatrix of*L(G)*calculated by discarding the column and row of u.*

Proof $\text{L}(G)=\left(\begin{array}{cc} d_{u} & -{\text{b}}^{⊤}\\ -\text{b} & {\text{L}}_{u} \end{array}\right)$ is the Laplacian matrix of *G*, where ${\text{L}}_{u}$ denotes the corresponding principal submatrix calculated by discarding the column and row of *u*. Because $\det(\text{L}(G))=\det({\text{L}}_{u})\det(d_{u}-{\text{b}}^{⊤}{\text{L}}_{u}^{-1}\text{b})=0$, we obtain $d_{u}={\text{b}}^{⊤}{\text{L}}_{u}^{-1}\text{b}$. Then

$$\begin{array}{c} \text{L}(G)=\left(\begin{array}{cc} 1 & \text{0}\\ {\text{L}}_{u}^{-1}\text{b} & \text{I} \end{array}\right)\left(\begin{array}{cc} 0 & -{\text{b}}^{⊤}\\ 0 & \text{R} \end{array}\right)\left(\begin{array}{cc} 1 & \text{0}\\ -{\text{L}}_{u}^{-1}\text{b} & \text{I} \end{array}\right), \end{array}$$

where

$$\text{R}={\text{L}}_{u}^{-1}\text{b}{\text{b}}^{⊤}+{\text{L}}_{u}.$$

Because R is nonsingular, we have the following,

$$\text{L}(G)=\left(\begin{array}{cc} 1 & \text{0}\\ {\text{L}}_{u}^{-1}\text{b} & \text{I} \end{array}\right)\left(\begin{array}{cc} 0 & -{\text{b}}^{⊤}\\ \text{0} & \text{R} \end{array}\right)\left(\begin{array}{cc} 1 & \text{0}\\ -{\text{L}}_{u}^{-1}\text{b} & \text{I} \end{array}\right)=\text{P}\left(\begin{array}{cc} 0 & \text{0}\\ \text{0} & \text{R} \end{array}\right){\text{P}}^{-1},$$

where

$$\text{P}=\left(\begin{array}{cc} 1 & \text{0}\\ {\text{L}}_{u}^{-1}\text{b} & \text{I} \end{array}\right)\left(\begin{array}{cc} 1 & -{\text{b}}^{⊤}{\text{R}}^{-1}\\ \text{0} & \text{I} \end{array}\right),\;{\text{P}}^{-1}=\left(\begin{array}{cc} 1 & {\;\text{b}}^{⊤}{\text{R}}^{-1}\\ \text{0} & \text{I} \end{array}\right)\left(\begin{array}{cc} 1 & \text{0}\\ -{\text{L}}_{u}^{-1}\text{b} & \text{I} \end{array}\right).$$

Let M=exp⁡(−tR), then

$$\begin{array}{cc} \exp(-t\text{L}(G)) & =\sum_{k=0}^{\infty }\frac{{\left(-t\right)}^{k}{\left(\text{L}(G)\right)}^{k}}{k!}=\sum_{k=0}^{\infty }\frac{{\left(\text{P}\left(\begin{array}{cc} 0 & \text{0}\\ \text{0} & -t\text{R} \end{array}\right){\text{P}}^{-1}\right)}^{k}}{k!}=\text{P}\left(\begin{array}{cc} 1 & \text{0}\\ \text{0} & \text{M} \end{array}\right){\text{P}}^{-1}\\ & =\left(\begin{array}{cc} 1 & \text{0}\\ {\text{L}}_{u}^{-1}\text{b} & \text{I} \end{array}\right)\left(\begin{array}{cc} 1 & -{\text{b}}^{⊤}{\text{R}}^{-1}\\ \text{0} & \text{I} \end{array}\right)\left(\begin{array}{cc} 1 & \text{0}\\ \text{0} & \text{M} \end{array}\right)\left(\begin{array}{cc} 1 & {\text{b}}^{⊤}{\text{R}}^{-1}\\ \text{0} & \text{I} \end{array}\right)\left(\begin{array}{cc} 1 & \text{0}\\ -{\text{L}}_{u}^{-1}\text{b} & \text{I} \end{array}\right)\\ & =\left(\begin{array}{cc} 1 & \text{0}\\ {\text{L}}_{u}^{-1}\text{b} & \text{I} \end{array}\right)\left(\begin{array}{cc} 1 & {\text{b}}^{⊤}{\text{R}}^{-1}(\text{I}-\text{M})\\ \text{0}\; & \text{M} \end{array}\right)\left(\begin{array}{cc} 1 & \text{0}\\ -{\text{L}}_{u}^{-1}\text{b} & \text{I} \end{array}\right)\\ & =\left(\begin{array}{cc} 1 & {\text{b}}^{⊤}{\text{R}}^{-1}(\text{I}-\text{M})\\ {\text{L}}_{u}^{-1}\text{b} & {\text{L}}_{u}^{-1}\text{b}{\text{b}}^{⊤}{\text{R}}^{-1}(\text{I}-\text{M})+\text{M} \end{array}\right)\left(\begin{array}{cc} 1 & \text{0}\\ -{\text{L}}_{u}^{-1}\text{b} & \text{I} \end{array}\right). \end{array}$$

As L(G)e=0, we obtain

$${\text{L}}_{u}\text{e}=\text{b},\;{\text{L}}_{u}^{-1}\text{b}=\text{e},\;\text{R}={\text{L}}_{u}^{-1}\text{b}{\text{b}}^{⊤}+{\text{L}}_{u}=(\text{J}+\text{I}){\text{L}}_{u},{\text{b}}^{⊤}{\text{R}}^{-1}={\text{b}}^{⊤}{\text{L}}_{u}^{-1}{\left(\text{J}+\text{I}\right)}^{-1}={\text{e}}^{⊤}{\left(\text{J}+\text{I}\right)}^{-1}=n^{-1}{\text{e}}^{⊤}.$$

Hence

$$\begin{array}{cc} \exp(-t\text{L}(G)) & =\left(\begin{array}{cc} 1 & n^{-1}{\text{e}}^{⊤}(\text{I}-\text{M})\\ \text{e} & n^{-1}\text{J}(\text{I}-\text{M})+\text{M} \end{array}\right)\left(\begin{array}{cc} 1 & \text{0}\\ -\text{e}\; & \text{I} \end{array}\right)\\ & =\left(\begin{array}{cc} 1+n^{-1}{\text{e}}^{⊤}(\text{M}-\text{I})\text{e} & n^{-1}{\text{e}}^{⊤}(\text{I}-\text{M})\\ \text{e}-n^{-1}\text{J}(\text{I}-\text{M})\text{e}-\text{M}\text{e} & n^{-1}\text{J}(\text{I}-\text{M})+\text{M} \end{array}\right). \end{array}$$

 □

We now obtain a reduction formula for calculating the diagonal entries of the HK using the principal submatrix of the Laplacian matrix. For graph *G*, these entries can be calculated according to Theorem 2.1.

Theorem 2.2 *Assume that G is a connected graph with n vertices. For any vertex*u,v∈V(G)*, we have*

$$H_{t}(u,u)={\left(\exp(-t\text{L}(G))\right)}_{uu}=n^{-1}+n^{-1}{\text{e}}^{⊤}\text{M}\text{e},H_{t}(v,v)={\left(\exp(-t\text{L}(G))\right)}_{vv}=n^{-1}+{\left(\text{M}\right)}_{vv}-n^{-1}c_{v}(\text{M})\;(v\neq u),$$

*where*$\text{M}=\exp(-t(\text{J}+\text{I}){\text{L}}_{u})$*,*${\text{L}}_{u}$*is the principal submatrix of*L(G)*calculated by discarding the column and row of u.*

The diagonal entries for the HK of a friendship graph are obtained as follows.

Example 2.3 Let $F_{n}$ denote a friendship graph with 2n+1 vertices. For $u,v\in V(G)\;(d_{u}\geq 3)$, then

$$H_{t}(u,u)={\left(\exp(-t\text{L}(G))\right)}_{uu}={\left(2n+1\right)}^{-1}+{\left(2n+1\right)}^{-1}{\text{e}}^{⊤}\exp(\text{Q})\text{e},H_{t}(v,v)={\left(\exp(-t\text{L}(G))\right)}_{vv}={\left(2n+1\right)}^{-1}+{\left(\exp(\text{Q})\right)}_{vv}-{\left(2n+1\right)}^{-1}c_{v}(\exp(\text{Q}))\;(v\neq u),$$

where $Q=-t\left(\begin{array}{cccc} \text{B} & \text{A} & \cdots & \text{A}\\ \text{A} & \text{B} & \cdots & \text{A}\\ \vdots & \vdots & \ddots & \vdots \\ \text{A} & \text{A} & \cdots & \text{B} \end{array}\right)$, $\text{B}=3{\text{I}}_{2}$, $\text{A}=\left(\begin{array}{cc} 1 & 1\\ 1 & 1 \end{array}\right)$.

Proof The diagonal entries of the HK are obtained via Theorem 2.2. □

For a graph *G* of order *n* with an almost equitable partition $V(G)=V_{1}\cup V_{2}\cup \cdots \cup V_{k}$, the corresponding characteristic matrix is an n×k matrix whose columns are the characteristic vectors of $V_{1},\ldots ,V_{k}$.

Next, we present the relationship between the Laplacian, generalized Laplacian, and characteristic matrices.

Lemma 2.4 *[23]**Let*L(G)*be the Laplacian matrix of graph G,*$\pi =\left(V_{1},\ldots ,V_{k}\right)$*be a k-partition of*V(G)*, and*${\text{P}}^{\pi }$*be a characteristic matrix of π. Then, π is an almost equitable k-partition if and only if there is a*k×k*-matrix*B*such that*

$$\text{L}(G){\text{P}}^{\pi }={\text{P}}^{\pi }\text{B}.$$

*Note that*B*is a generalized Laplacian matrix*${\text{L}}^{\pi }$*when π is an almost equitable k-partition.*

Next, we formulate the HK diagonal entries via almost equitable partitions of graphs, diminishing the diagonal entries of the HK of *G* with a smaller matrix.

Theorem 2.5 *Assuming that G has an almost equitable partition* $V(G)=V_{1}\cup V_{2}\cup \cdots \cup V_{k}$ *and* $V_{1}=\{u\}$ *for vertex* u∈V(G) *, then*

$$H_{t}(u,u)={\left(\exp\left(-t\text{B}\right)\right)}_{V_{1}V_{1}},$$

*where B is the generalized Laplacian matrix of the almost equitable partition.*

Proof Assuming that C and B are the characteristic and generalized Laplacian matrices of an almost equitable partition, respectively, Lemma 2.4 asserts the following,

$$\exp\left(-t\text{L}\left(G\right)\right)\text{C}=\sum_{k=0}^{\infty }\frac{1}{k!}{\left(-t\right)}^{k}{\left(\text{L}\left(G\right)\right)}^{k}\text{C}=\text{C}\sum_{k=0}^{\infty }\frac{1}{k!}{\left(-t\right)}^{k}{\text{B}}^{k}=\text{C}\exp\left(-t\text{B}\right).$$

As $V_{1}=\{u\}$, we obtain

$${\left(\exp\left(-t\text{L}\left(G\right)\right)\right)}_{uu}={\left(\exp\left(-tL\left(G\right)\right)\text{C}\right)}_{uV_{1}}={\left(\text{C}\exp\left(-t\text{B}\right)\right)}_{uV_{1}}={\left(\exp\left(-t\text{B}\right)\right)}_{V_{1}V_{1}}.$$

 □

Combining $G_{1}$ and $G_{2}$ yields a graph obtained from $G_{1}\cup G_{2}$ by joining each vertex in $G_{1}$ to that in $G_{2}$. If $G_{2}$ is an isolated vertex, the output from joining $G_{1}$ and $G_{2}$ is a cone over $G_{1}$ [22].

Corollary 2.6 *Assuming that u is a newly added vertex in H, where H is the cone over a regular graph G with n vertices, the diagonal entries of u in H corresponding to the heat kernel are*

$$\begin{array}{c} H_{t}(H)(u,u)=\frac{-1+n\exp(-t(n-1))}{n-1}. \end{array}$$

Proof Because *H* has an almost equitable partition V(H)={u}∪V(G), the corresponding generalized Laplacian matrix is $\text{B}=\left(\begin{array}{cc} n\; & -n\\ 1\; & -1 \end{array}\right)$, which is also the Laplacian matrix of a quotient graph corresponding to *G*. Let

$$\begin{array}{c} \text{P}=\left(\begin{array}{cc} 1 & n\\ 1 & 1 \end{array}\right), \end{array}$$

then

$$\begin{array}{c} {\text{P}}^{-1}=\left(\begin{array}{cc} -\frac{1}{-1+n} & \frac{n}{-1+n}\\ \frac{1}{-1+n} & -\frac{1}{-1+n} \end{array}\right). \end{array}$$

So, we have

$$\begin{array}{c} \text{B}=\text{P}\left(\begin{array}{cc} 0 & 0\\ 0 & n-1 \end{array}\right){\text{P}}^{-1}. \end{array}$$

$$\begin{array}{cc} H_{t}(\overset{˜}{G})=\exp(-t\text{B}) & =\text{P}\left(\begin{array}{cc} 1 & 0\\ 0 & \exp(-t(n-1)) \end{array}\right){\text{P}}^{-1}\\ & =\left(\begin{array}{cc} 1 & n\\ 1 & 1 \end{array}\right)\left(\begin{array}{cc} 1 & 0\\ 0 & \exp(-t(n-1)) \end{array}\right)\left(\begin{array}{cc} -\frac{1}{-1+n} & \frac{n}{-1+n}\\ \frac{1}{-1+n} & -\frac{1}{-1+n} \end{array}\right)\\ & =\left(\begin{array}{cc} \frac{-1+n\exp(-t(n-1))}{n-1} & \frac{n-n\exp(-t(n-1))}{n-1}\\ \frac{-1+\exp(-t(n-1))}{n-1} & \frac{n-\exp(-t(n-1))}{n-1} \end{array}\right). \end{array}$$

Theorem 2.5 states the following,

$$\begin{array}{c} H_{t}(H)(u,u)=\exp{\left(-t\text{B}\right)}_{11}=\frac{-1+n\exp(-t(n-1))}{n-1}. \end{array}$$

 □

We now employ Corollary 2.6 to calculate all diagonal entries of the HK for a wheel graph.

Example 2.7 Assuming that the wheel graph $W_{n+1}$ represents the join of $C_{n}$ and $K_{1}$, where $C_{n}$ is a cycle of order *n* and $K_{1}$ is an isolated vertex, $W_{n+1}$ represents the cone over of cycle graph $C_{n}$. The Laplacian spectra of $W_{n+1}$ are $3-2\cos\frac{2\pi i}{n}(i=1,2,\ldots ,n-1)$, 0 and n+1 (see [28]). According to Corollary 2.6, the HK diagonal entries of the vertex of degree *n* in $W_{n+1}$ are

$$\frac{-1+(n+1)\exp(-tn)}{n}.$$

The HK trace of $W_{n+1}$ is

$$\mathrm{Tr}\left(H_{t}\left(W_{n+1}\right)\right)=\sum_{i=1}^{n-1}\exp\left(-3t+2t\cos\frac{2\pi i}{n}\right)+\exp\left(-nt-t\right)+1.$$

Because all vertices of degree 3 in $W_{n+1}$ have the same HK diagonal entries, we can conclude that the HK diagonal entries of a vertex of degree 3 in $W_{n+1}$ are

$$\frac{n\sum_{i=1}^{n-1}\exp\left(-3t+2t\cos\frac{2\pi i}{n}t\right)+n\exp\left(-nt-t\right)+n+1-(n+1)\exp(-tn)}{n^{2}}.$$

Remark 1 For further research on the Laplacian spectra of cones over regular graphs (e.g., $sC_{m}\cup K_{1}$ and $sK_{m}\cup K_{1}$), refer to [29]). The HK diagonal entries of these composite graphs (networks) can be obtained directly using Corollary 2.6.

## 3 Bounds of heat kernel trace for graphs

We now obtain an approximation of the HK trace for *G*.

Lemma 3.1 *[22]* *Assuming that G is a graph with m edges and n vertices, then*

$$\begin{array}{cc} \mathrm{Tr}\left(\text{L}(G)\right)= & \sum_{i=1}^{n}\lambda_{i}=2m\\ \mathrm{Tr}\left({\text{L}}^{2}(G)\right)= & \sum_{i=1}^{n}\lambda_{i}^{2}=2m+\sum_{i=1}^{n}d_{i}^{2}. \end{array}$$

Lemma 3.2 *[30]* *Assuming that G is a graph with m edges and n vertices, then*

$$\begin{array}{c} \sum_{i=1}^{n}d_{i}^{2}\leq m\left(\frac{2m}{n-1}+n-2\right). \end{array}$$

We now obtain the upper and lower bounds of HE(G) using the vertex and edge numbers.

Theorem 3.3 *Assuming that G is a graph with m edges and n vertices, then*

$$\sqrt{n-4tm+n(n-1)e^{-\frac{4tm}{n}}}\leq HE(G)\leq n-2tm-1-\omega +e^{\omega },$$

*where*$\omega =\sqrt{2mt^{2}+mt^{2}\left(\frac{2m}{n-1}+n-2\right)}$*and the above equality holds if and only if*$G≅\bar{K_{n}}$*.*

Proof $$\begin{array}{c} HE^{2}(G)=\sum_{i=1}^{n}e^{-2t\lambda_{i}}+2\sum_{1\leq i<j\leq n}e^{-t\lambda_{i}}e^{-t\lambda_{j}}. \end{array}$$

From the arithmetic-geometric inequality, we have

$$\begin{array}{cc} 2\sum_{1\leq i<j\leq n}e^{-t\lambda_{i}}e^{-t\lambda_{j}}\geq & n(n-1){\left(\prod_{1\leq i<j\leq n}e^{-t\lambda_{i}}e^{-t\lambda_{j}}\right)}^{\frac{2}{n(n-1)}}\\ = & n(n-1){\left(\prod_{1\leq i\leq n}^{n}{\left(e^{-t\lambda_{i}}\right)}^{n-1}\right)}^{\frac{2}{n(n-1)}}\\ = & n(n-1){\left(\prod_{1\leq i\leq n}^{n}e^{-t\lambda_{i}}\right)}^{\frac{2}{n}}\\ = & n(n-1){\left(e^{-t\sum_{i=1}^{n}\lambda_{i}}\right)}^{\frac{2}{n}}\\ = & n(n-1)e^{-\frac{4tm}{n}}. \end{array}$$

From the Taylor expansion, we obtain

$$\begin{array}{cc} \sum_{i=1}^{n}e^{-2t\lambda_{i}}= & \sum_{i=1}^{n}\sum_{k=0}^{\infty }\frac{{\left(-2t\lambda_{i}\right)}^{k}}{k!}=n-4tm+\sum_{i=1}^{n}\sum_{k=2}^{\infty }\frac{{\left(-2t\lambda_{i}\right)}^{k}}{k!}. \end{array}$$

Because $e^{-2t\lambda_{i}}\geq 1-2t\lambda_{i}$ holds for all *i*, $\sum_{k=2}^{\infty }\frac{{\left(-2t\lambda_{i}\right)}^{k}}{k!}\geq 0$. Then

$$\begin{array}{cc} \sum_{i=1}^{n}e^{-2t\lambda_{i}}= & n-4tm+\sum_{i=1}^{n}\sum_{k=2}^{\infty }\frac{{\left(-2t\lambda_{i}\right)}^{k}}{k!}\\ \geq & n-4tm. \end{array}$$

Substituting the above formula and solving for HE(G), we obtain

$$HE^{2}(G)\geq n-4tm+n(n-1)e^{-\frac{4tm}{n}},$$

then

$$HE(G)\geq \sqrt{n-4tm+n(n-1)e^{-\frac{4tm}{n}}},$$

where t>0.

By Lemma 3.1, Lemma 3.2, we obtain an upper bound.

$$\begin{array}{cc} HE(G)= & n-2tm+\sum_{i=1}^{n}\sum_{k=2}^{\infty }\frac{{\left(-t\lambda_{i}\right)}^{k}}{k!}\\ \leq & n-2tm+\sum_{i=1}^{n}\sum_{k=2}^{\infty }\frac{|-t\lambda_{i}{|}^{k}}{k!}\\ = & n-2tm+\sum_{k=2}^{\infty }\frac{1}{k!}\sum_{i=1}^{n}|t\lambda_{i}{|}^{k}\\ \leq & n-2tm+\sum_{k=2}^{\infty }\frac{1}{k!}{\left(\sum_{i=1}^{n}t^{2}\lambda_{i}^{2}\right)}^{k/2}\\ \leq & n-2tm+\sum_{k=2}^{\infty }\frac{1}{k!}{\left(2mt^{2}+mt^{2}\left(\frac{2m}{n-1}+n-2\right)\right)}^{k/2}\\ = & n-2tm-1-\omega +\sum_{k=0}^{\infty }\frac{\omega^{k}}{k!}\\ = & n-2tm-1-\omega +e^{\omega }, \end{array}$$

where $\omega =\sqrt{2mt^{2}+mt^{2}\left(\frac{2m}{n-1}+n-2\right)}$.

From the above derivation, it is evident that equality is attained if and only if graph *G* has all zero eigenvalues, which occurs only in the case of the edgeless graph $\bar{K_{n}}$. □

## 4 Concluding remarks

Because a given graph *G* contains almost equitable partitions and a principal submatrix L(G), we derived new equations for the HK diagonal entries of *G* in the context of these features. Compared to the Laplacian matrix of *G*, the derived formulas employ smaller matrices to obtain the aforementioned diagonal entries. Furthermore, we obtained certain bounds for the HK trace. Our future work will focus on HK diagonal entry formulas for digraphs, as well as the approximation of the HK trace using other graph parameters.

## Funding statement

Hebei Province high-level talent funding project (B20221014, C20221079).

## Additional information

No additional information is available for this paper.

## CRediT authorship contribution statement

**Yang Yang:** Writing – review & editing, Writing – original draft, Visualization, Validation, Supervision, Software, Resources, Project administration, Methodology, Investigation, Formal analysis, Data curation, Conceptualization. **Wei Ke:** Writing – review & editing, Funding acquisition. **Zhe Wang:** Writing – review & editing, Funding acquisition. **Haiyan Qiao:** Writing – review & editing, Writing – original draft, Visualization, Validation, Supervision, Software, Resources, Project administration, Methodology, Investigation, Funding acquisition, Formal analysis, Data curation, Conceptualization.

## Declaration of Competing Interest

The authors declare that they have no known competing financial interests or personal relationships that could have appeared to influence the work reported in this paper.

## Data Availability

No data was used for the research described in the article.
